# Poly-ADP Ribosyl Polymerase 1 (PARP1) Regulates Influenza A Virus Polymerase

**DOI:** 10.1155/2019/8512363

**Published:** 2019-03-19

**Authors:** Liset Westera, Alisha M. Jennings, Jad Maamary, Martin Schwemmle, Adolfo García-Sastre, Eric Bortz

**Affiliations:** ^1^Department of Microbiology, Icahn School of Medicine at Mount Sinai, New York, New York, USA; ^2^Department of Biological Sciences, University of Alaska Anchorage, Anchorage, Alaska, USA; ^3^Institute of Virology, University Medical Center Freiburg, 79104 Freiburg, Germany

## Abstract

Influenza A viruses (IAV) are evolutionarily successful pathogens, capable of infecting a number of avian and mammalian species and responsible for pandemic and seasonal epidemic disease in humans. To infect new species, IAV typically must overcome a number of species barriers to entry, replication, and egress, even while virus replication is counteracted by antiviral host factors and innate immune mechanisms. A number of host factors have been found to regulate the replication of IAV by interacting with the viral RNA-dependent RNA polymerase (RdRP). The host factor PARP1, a poly-ADP ribosyl polymerase, was required for optimal functions of human, swine, and avian influenza RdRP in human 293T cells. In IAV infection, PARP1 was required for efficient synthesis of viral nucleoprotein (NP) in human lung A549 cells. Intriguingly, pharmacological inhibition of PARP1 enzymatic activity (PARylation) by 4-amino-1,8-naphthalimide led to a 4-fold increase in RdRP activity, and a 2.3-fold increase in virus titer. Exogenous expression of the natural PARylation inhibitor PARG also enhanced RdRP activity. These data suggest a virus-host interaction dynamic where PARP1 protein itself is required, but cellular PARylation has a distinct suppressive modality, on influenza A viral polymerase activity in human cells.

## 1. Introduction

Influenza viruses are segmented, negative-sense single-stranded RNA viruses in the evolutionarily diverse viral family Orthomyxoviridae. Annual seasonal epidemics of influenza A virus (IAV) infections are a considerable health burden in humans. The natural reservoir of IAV is in wild birds, although AIV can infect poultry, and highly pathogenic avian influenza viruses (HPAIV) of H5N1, H5N6, and H7N9 hemagglutinin subtypes, among others, can spread to humans [1, 2]. Epizootic IAV infections occur frequently in seals, horses, and swine [1, 3–5]. Reassortment of the 8 viral gene segments in IAV genomes can result in emergence of immunologically distinct strains, capable of rapid, virulent spread in susceptible populations, gravely illustrated by the high burden of influenza and mortality of the 1918 H1N1 pandemic in humans [6]. In 2009, a novel reassortant strain of IAV (pdmH1N1), in part a genetic descendant of the 1918 H1N1 strain, swine, and avian viruses [7], emerged from swine to spread globally in humans again, causing considerable respiratory disease, particularly in patients with underlying medical conditions [8]. The pdmH1N1 pandemic virus also spread to other species, including elephant seals, and contributed gene segments to novel strains in swine (H3N2v) that are capable of infecting humans [9–11]. Thus, although restricted by natural or vaccine-generated subtype-specific immune responses against surface hemagglutinin (HA) and neuraminidase (NA) proteins, as a virus group, IAV has proved capable of overcoming host barriers to achieve replication in multiple species [1, 2, 12]. This suggests that the fundamental processes of the IAV life cycle, including HA-dependent binding and entry into a susceptible host cell, viral RNA and protein syntheses, virion assembly, and NA-dependent maturation, can utilize host cell molecular structures and physiological processes that are broadly conserved in multicellular vertebrates.

The IAV RNA-dependent RNA polymerase (RdRP) is a critical determinant of viral pathogenesis and transmission of IAV from avian to mammalian species [7, 13–15]. The IAV RdRP, consisting of heterotrimeric PB1, PB2, and PA proteins, in conjunction with viral nucleoprotein (NP), functions as both a transcriptase that binds viral (-) sense vRNA genomes to synthesize viral mRNA, and as a replicase that synthesizes vRNA progeny via a (+) sense cRNA intermediate [16]. Importantly, these processes are modulated by interacting cellular proteins. Critically, host RNA polymerase II aids in initiation, cap-snatching, and elongation of viral mRNA syntheses during transcription [17–19]. A large number of other host cell proteins interact with the IAV RdRP complex in nuclei of infected cells [20–22]. Experiments targeting host genes by RNA interference (RNAi) or CRISPR knockout [23] showed that a number of cellular factors are required for IAV infection. Among these are host factors regulating the viral polymerase, such as the RNA binding protein DDX17 [24], DDX19 [25] protein kinase C [26], and ANP32A/pp32 [27]. Alternately, antiviral factors, such as NF90 [28] and MXA [29], antagonize RdRP functions. Influenza RdRP activity is typically examined in cell culture infection by cotransfection of luciferase reporters and plasmids encoding PB1, PB2, PA, and NP, in an optimized viral polymerase minigenome reporter assay and by primer extension for detection of viral RNA species [24].

The poly-ADP ribosyl polymerase 1 (PARP1) protein was identified as an interacting partner of influenza A virus polymerases [20]. Poly-ADP ribosyl polymerases contain an enzymatic active site domain that adds ADP ribosyl polymeric oligonucleotides to target molecules (PARylation). ADP ribosyl polymers affect the activity of a number of proteins, in turn modulating cellular pathways including cell cycle, DNA replication, apoptosis, and metabolic cell viability [30]. PARP1 restricts replication of Kaposi's sarcoma-associate herpesvirus (KSHV/HHV-8) by PARylation of the KSHV immediate-early transactivator RTA, but is blocked by viral processivity factor PF-8 [31]. Other PARP family members, including PARP7, PARP10, and PARP12L, are interferon- (IFN-) induced proteins that have antiviral functions by limiting translation [32]. Interestingly, complete CRISPR/Cas9 knockout of PARP1 leads to induction of type I IFN, possibly due to aberrant cellular RNA species [33] and PARP12 activation [34]. Isoforms of PARP13 are antiviral [35], including ZAPS which activates RIG-I [36], while ZAPL specifically disrupts the PB2 and PA proteins of the influenza RdRP complex, but is counteracted by the viral PB1 protein [37].

Our previous work has suggested that PARP1 is required for IAV replication [24]; however, the mechanism of this regulation is poorly understood. We observed that PARP1 has been found to interact with a network of cellular transcriptional regulatory proteins that also modulated IAV infection, including NCL, NPM1, DDX21 [38], HSP90 [39], RBM14, and the DNA-PK/Ku70/Ku86 (XRCC1/5/6) complex [24]. Experiments in cell culture infections showed a >50% reduction in infectious IAV titer released from cells depleted of PARP1 by RNAi [20, 24]. We also showed that PARP1 is specifically required for the activity of the viral RdRP of human H1N1 and avian-derived H5N1 viruses [24], although the mechanism of this requirement was unknown.

However, the* PARP1 *homozygous null (-/-) mouse exhibits accelerated aging and spontaneous tumorigenesis [40], and human HCT116 colon carcinoma cells that are deficient in both* PARP1* alleles by CRISPR/Cas9 knockout exhibited reduced growth rates, increased cellular senescence and DNA-damage, and aberrant interferon responses [33]. These findings highlight the importance of ablating PARP1 by other less drastic methods such as partial disruption of PARP1 protein synthesis by RNAi knockdown targeting* PARP1* mRNA [41] and inhibition of PARP's PARylation activity with small molecule inhibitors [42]. To understand how PARP1 modulates influenza virus life cycle, we studied the relationship between PARP1, cellular PARylation, and activity of the IAV RdRP. We found that the dependence of viral polymerase activity on poly-ADP ribosyl polymerase and its enzymatic activity is complex, reflecting the many roles of PARP1 in cellular physiology.

## 2. Materials and Methods

### 2.1. Cell Cultures, Drug Treatments, and Viruses

Human embryonic kidney (HEK 293T) fibroblasts, human lung adenocarcinoma (A549) cells, and Madin-Darby Canine Kidney (MDCK) cells were cultured at 37°C in a 5% CO2 atmosphere in DMEM (Corning Inc., Manassas, VA), supplemented with 10% FBS (Atlas Biologicals, Fort Collins, CO) and antibiotics (1X penicillin/streptomycin). Cells were treated with pharmacological agents, doxorubicin or 4-amino-1,8-naphthalimide (4-AN), obtained from Sigma-Aldrich (St. Louis, MO), after determination of subtoxic dose (1-20uM) by measurement of A549 cell viability using CellTiter-Glo and Caspase-3/7 Glo assays (Promega, Madison WI). Influenza A/WSN/1933 (H1N1) and A/PR/8/34 (H1N1) viruses were grown as described previously [24, 43]. All assays were performed on biological duplicate wells in culture, with dual readings per well (4 measurements per condition). Low-path (attenuated) avian-derived influenza A/Viet Nam/1203/2004 (H5N1) HALo virus was generated by reverse genetics with removal of the hemagglutinin (HA0) protein's polybasic cleavage site (GenBank Accession no. CY077101); the virus undergoes only one round of replication in the absence of exogenous trypsin. All other wild-type viral gene segments were unmodified. As this virus plaques poorly in absence of exogenous trypsin, titers of low-path, avian-derived H5N1 HALo influenza viruses were determined by limiting-dilution immunofluorescence assay on A549 cells, with NP-staining cells counted in triplicate for titration, averaged from tenfold dilutions. All experiments with live H5N1 HALo virus were conducted at the Icahn School of Medicine at Mount Sinai, under biosafety regimen described previously [24], with review of protocols under a Dual-Use Research of Concern (DURC) framework.

### 2.2. RNA Interference Experiments

Small interfering RNA (siRNA) pools containing 1-4 distinct siRNA (Ambion/Life Technologies, Carlsbad, CA; Dharmacon, Lafayette, CO) were used to target and knock down host factor transcripts, including PARP1, NPM, DDX17, IMPDH2, and Ku70/Ku86 (simultaneously), and compared to nontarget (scrambled) siRNA, as previously described [24, 43]. Briefly, siRNA (10-15nM) were transfected into cells using Lipofectamine 2000 (Life Technologies, Carlsbad, CA) 24-36 hours prior to transfection of viral polymerase reporter cDNA for minigenome experiments in 293T cells or infection of A549 cells with influenza viruses. All assays were performed on biological duplicate wells in culture, with dual readings per well (4 measurements per condition). The effect of siRNA transfection on cell viability was measured by CellTiter-Glo and Caspase-3/7 Glo assays, and target gene knockdown efficiency was validated by quantitative RT-PCR or Western blotting, as available, or as described previously [24].

### 2.3. Minigenome Reporter Assays

To assay IAV RdRP activity, an optimized polymerase minigenome luciferase reporter assay was used, as described previously [24]. Briefly, in minigenome reporter assays, viral polymerase, and NP plasmids (total 375ng), 100ng of a vRNA-promoter reporter encoding firefly luciferase and 25ng of a constitutive* Renilla* luciferase internal control plasmid (with a cellular pol II-driven actin promoter ad CME immediate-early enhancer element) were cotransfected into cells in 24-well format using Lipofectamine 2000. Optimized plasmid (ng) ratios of 10:2:1:2 (NP:PB1:PB2:PA), or 5:2:1:2 for experiments adding exogenous cDNA of host factors (vector, PARP1, PARG, NPM1, or IMPDH2) were determined experimentally to maximize luciferase RLU/ng plasmid. Dual luciferase assay (DLR; Promega) was used to measure polymerase activity. The vRNA reporter encoding firefly luciferase alone (100ng) was transfected into A549 cells for assessment of authentic, infected cell IAV RdRP activity [44], one day prior infection, and analyzed for luciferase assay after 20 hours after infection. All assays were performed on biological duplicate wells in culture, with dual readings per well (4 measurements per condition). Staurosporine cotreatment (1uM) induced 78-108-fold higher Caspase-3/7 Glo assay (Promega) than mock indicating apoptosis induction.

### 2.4. PARylation Assay

Total cellular poly-ADP ribosyl polymerization (PARylation the enzymatic activity of PARP proteins) was analyzed by HT chemiluminescent PARP/Apoptosis Assay kit (Trevigen, Gaithersburg, MD) and was measured from A549 cell extracts duplicated for each experimental condition, according to the manufacturer's instructions. HRP chemiluminescence of PARylation of a histone substrate was measured in a BioTek Synergy HT plate reader set on the luciferase channel, with averaged results of two readings (gain=80 and gain=100) for each condition compared to a standard curve. An unpaired, 2-tailed t-test was used to estimate a statistical significance (*p* value).

### 2.5. Other Analyses of IAV Infection in Cells

Primer extension assay was performed as previously described [24]; statistical analysis of primer extension data was performed by one-way ANOVA, analyzing normalized band density readings (density histograms), in comparisons to background and housekeeping RNA bands (5S rRNA) across all conditions and within each condition; significance of differences in normalized band density were estimated by unpaired, 2-tailed t-test. Immunoprecipitation of virus and host proteins from transfected 293T cells used M2 (FLAG) agarose beads (Sigma), and probing on Western blot with specific antibodies [20]; RNase was from Qiagen. Transfection of cDNA encoding host factors using Lipofectamine 2000, and immunofluorescence microscopy and Western blotting were performed as described previously [24].

## 3. Results

### 3.1. PARP1 Is Required for Influenza A Virus RNA-Dependent RNA Polymerase Function

**Figure 1:**
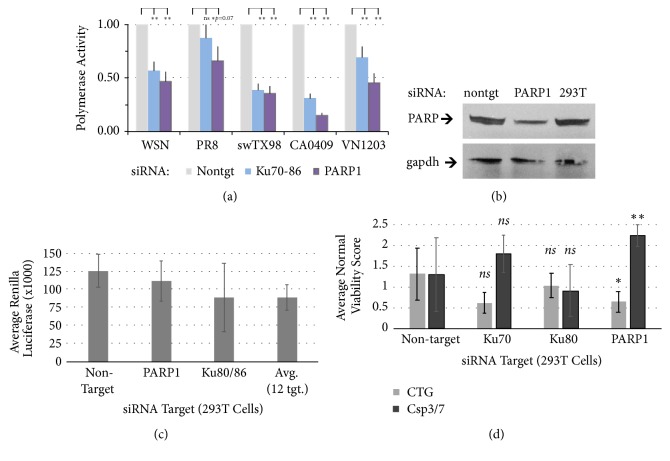
PARP1 is required for optimal activity of the influenza A virus RNA-dependent RNA polymerase. (a)* Host factors are required for influenza A virus polymerase function. *For minigenome reporter assays, cDNA encoding influenza polymerase proteins (PB1, PB2, PA, and NP), a firefly luciferase reporter driven by a virus RdRP-binding site promoter, and a constitutive* Renilla* luciferase internal reference were transfected into human HEK 293T cells targeted with siRNA against human PARP1, Ku70, and Ku80/86 (Ku70-86), or scrambled siRNA control (Nontgt), in duplicate. Polymerases from influenza A virus strains included human A/WSN/33 (H1N1)* (WSN)*, human A/PR/8/34 (H1N1)* (PR8)*, A/swine/Texas/4199-2/98 (H3N2)* (swTX98)*, human A/California/04/09 (pdmH1N1)* (CA0409)*, or avian-derived A/Viet Nam/1203/04 (H5N1)* (VN1203)*. Polymerase activity of the negative control was normalized for each strain to 1.0. (b) Immunoblot showing PARP1 protein depletion with GAPDH protein as internal reference. Cellular RNA polymerase II-mediated expression of plasmid encoding* Renilla* luciferase (c) and (d) cell viability assays measuring ATP availability (CellTiter-Glo,* CTG*) and apoptosis by caspase activation (*Csp3/7*), in 293T cells targeted with siRNA. Significance estimated by 2-tailed t-test, with* p *values* p<*0.05 (*∗∗*) or* p<*0.1 (*∗*) indicated.

Our earlier studies indicated that PARP1 interacts with and is required for activity of the influenza RNA-dependent RNA polymerase (RdRP) [20, 24]. These experiments showed a reduction in polymerase activity during infection by H1N1 (57%) and avian H5N1 (83%) viruses [24]. Therefore, to further study IAV polymerase genotypes more comprehensively, we examined the requirement for PARP1 for activity of the polymerase for human, swine, and avian-derived influenza strains using an optimized influenza polymerase minigenome reporter assay. To this end we compared knockdown of the requirement for PARP1 to knockdown of the DNA damage repair complex proteins Ku70/86 that were also found to be required host factors for the influenza replication and polymerase function [24] and interact with PARP1 in DNA-damage repair (DDR) proteome network [45]. Similar to Ku70/86, PARP1 was required for optimal activity of polymerases from five different influenza strains (Figure 1(a)): seasonal human H1N1 derived from the 1918 pandemic H1N1 (WSN and PR8), the polymerase from the swine triple reassortant (TRIG) A/swine/Texas/4199-2/98 (H3N2) [46], the human pandemic 2009 H1N1 polymerase A/California/04/2007 (pdmH1N1) [44, 47], and avian-derived A/Viet Nam/1203/2004 (H5N1) polymerase from a fatal human case of highly pathogenic avian influenza [48]. The degree of dependence of the influenza polymerases on PARP1 was generally over 2-fold and varied among strains (34-85% of polymerase activity, average 58%) and was significant (*p*<0.05). The human pandemic 2009 H1N1 polymerase A/California/04/2007 (pdmH1N1) polymerase exhibited over 4-fold reduction in activity with the exception of PR8 that was not indicated as significant dependence on PARP1 in this assay (*p*=0.07). Knockdown of PARP1 by siRNA in this assay leads to an observable decrease in PARP1 protein in the cells; however, depletion is not complete (Figure 1(b)). PARP1 or Ku70/86 knockdown did not affect cellular RNA polymerase II-mediated gene expression (Figure 1(c)), but PARP1 knockdown did weakly induce loss of ATP activity (*p*=0.06) and induction of caspase-3/7 cleavage indicating initiation of apoptotic pathways (*p*=0.04) (Figure 1(d)). Thus, it is possible that the residual PARP1 provides functions required for a limited degree of influenza polymerase activity and maintenance of cellular viability.

### 3.2. PARP1 Is Required for Synthesis of Viral RNA and NP during Infection

To better understand the mechanism by which PARP1 regulates influenza polymerase activity, we examined PARP1's role in synthesis of viral mRNA and vRNA species by primer extension assay in human 293T cells targeted by siRNA against* PARP1* and infected with low-path H5N1 HALo virus. Although the magnitude of differences was not very large, with only ~10% reduction in viral RNA syntheses, a weak trend (*p<*0.09) where PARP1 was required for synthesis of viral mRNA and vRNA encoding NP and HA was observed (Figure S1). It should be noted that siRNA-mediated knockdown only mildly depleted PARP1 protein (Figure S1B) in this assay.

**Figure 2:**
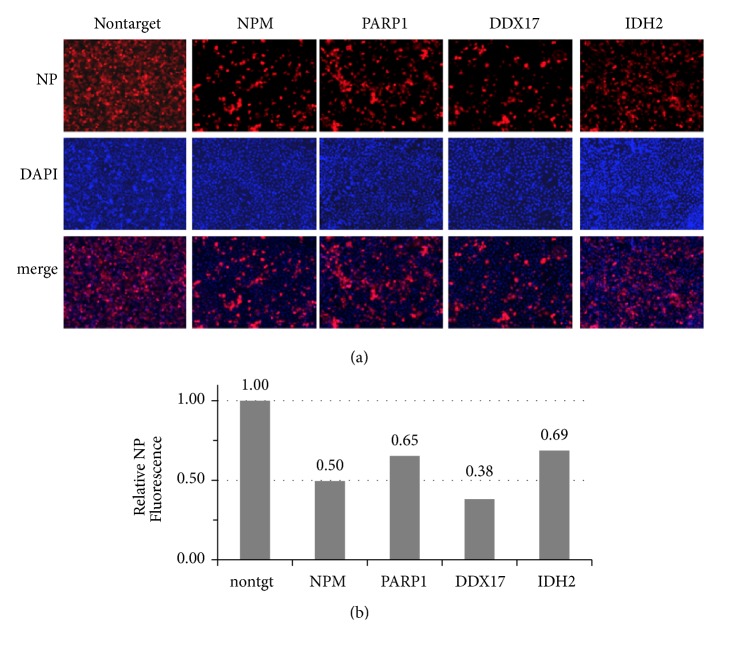
PARP1 is required for synthesis of influenza nucleoprotein. (a) Host factors were targeted by RNA interference in human lung A549 cells, and after 36h knockdown, cells were infected with low-path, avian-derived influenza virus strain A/Viet Nam/1203/04 (H5N1) HALo (MOI = 0.1). Host factors targets: nucleophosmin 1 (NPM), PARP1, DEAD-box RNA helicase 17 (DDX17), and inosine monophosphate dehydrogenase 2 (IMPDH2, here shortened to IDH2), in comparison to scrambled negative control siRNA (nontarget). (b) Mean, normalized relative fluorescence intensity of total NP immunofluorescence in images quantified by (Alexa 555nm) fluorescent microscopy. siRNA targets are shown on horizontal axis.

Thus, we next sought to understand how PARP1 affected synthesis of viral nucleoprotein (NP). When PARP1 was targeted by siRNA, expression of viral NP during infection of human lung A549 cells with low-path, avian-derived influenza virus strain A/Viet Nam/1203/04 (H5N1) HALo was considerably reduced (Figure 2(a)). NP accumulation was reduced similar to knockdown of RNA binding proteins NPM1 and DDX17 that are known to be required for influenza virus polymerase activity [24]. The ribonucleotide synthesis enzyme inosine monophosphate dehydrogenase 2 (IMPDH2), a cellular factor that interacts with the PARP1 and PARP2 DDR proteome network [45, 49], was also required for influenza NP synthesis. Further study of PARP1 role in kinetics of viral RNA syntheses and expression of other viral proteins will require generation of viable knockout (CRISPR/Cas9) cell lines in* PARP1 *[23].

### 3.3. Inhibition of PARP Enzyme PARylation Enhances Activity of the Influenza Polymerase

**Figure 3:**
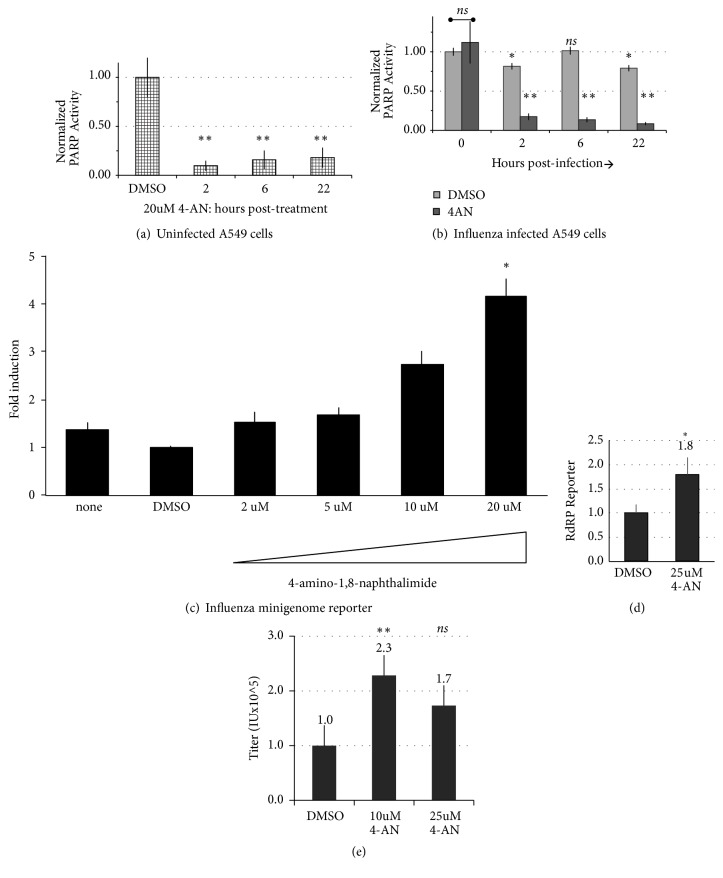
*Inhibitor of PARP1/2 proteins PARylation activity increases IAV RDRP activity*. (a) Assay of cellular PARylation after treatment by 4-amino-1,8-naphthalimide (4-AN) for 20h in A549 cells. (b) Poly-ADP ribosylation (PARP) enzymatic activity was analyzed in protein lysates from A549 cells treated with DMSO vehicle, 20uM 4-AN, or 1uM doxorubicin (DOXO), and infected 20h with IAV (A/PR/8/34 H1N1, MOI = 1); lysates were analyzed by PARylation assay. (c) HEK 293T cells were transfected with NP and polymerase cDNA plasmids in IAV minigenome reporter assay and untreated, treated with vehicle (DMSO), or increasing doses of PARP inhibitor drug (4-AN). (d) HEK 293T cells were transfected with RdRP firefly luciferase reporter construct one day prior vehicle (DMSO) or treatment with 25uM 4-AN and infected with low-path, H5N1 HALo virus (MOI = 1). Infected cell viral polymerase activity was analyzed after 20h by luciferase assay; two-tailed t-test (*∗*)* p*=0.06. (e) Single-step growth of H5N1 HALo virus (MOI = 1) in A549 cells pretreated with 4-AN or vehicle (DMSO) as indicated. Infectious titer (IU) released 1d.p.i. measured by limiting dilution assay for NP-positive cells. Two-tailed t-test for significance in comparison to vehicle: *∗∗*,* p*<0.05;* ns,* not significant. All experiments were performed in biological duplicates with two readings per well.

As poly-ADP ribosyl polymerases encode an enzymatic activity that adds polymeric ADP ribosyl oligonucleotides to target molecules, affecting numerous cellular transcriptional processes, we investigated the role of this enzymatic activity in influenza infection. To assess the requirement of the enzymatic activity of PARP1 in influenza RdRP function, we directly inhibited PARylation using small molecule inhibitor 4-amino-1,8-naphthalimide (4-AN). According to recent studies, the vast majority of cellular PARylation activity is catalyzed by PARP1 (85%-90%) with the remainder mostly by PARP2 [30]. The drug 4-AN inhibits the enzymatic activity of both PARP1 and PARP2, the most abundant active PARP enzymes. We measured PARylation activity of total A549 cell extracts by* in vitro* PARylation of a histone substrate, essentially measuring the total activity of PARP1, PARP2, and other PARP enzymes. Subtoxic treatment with 4-AN (20uM) effectively reduced total cell PARylation by approximately 90% within two hours of treatment, with a weak recovery (to <20%) after 1 day of treatment (Figure 3(a)). Next, total PARylation activity was assessed in cells over the course of influenza A virus life cycle. In A549 cells infected with influenza A/WSN/33 (H1N1) virus (MOI = 1), PARylation activity was relatively stable over the course of infection (Figure 3(b)). A mild, transient loss of PARylation activity at early timepoints was not significant, as variation in cellular PARP1 protein abundance was evident through the course of infection (Figure S2). However, influenza infection itself did not alter cellular PARylation, suggesting that the activity of PARP1, PARP2, and other PARP enzymes is not significantly targeted by viral proteins. This contrasted with treatment with the specific drug inhibitor 4-AN that drastically reduced PARylation activity in infected cells (Figure 3(b)) as it does in uninfected cells (Figure 3(a)).

As RNAi knockdown experiments showed that PARP1 is required for influenza polymerase function and virus replication, we next sought to examine the influenza polymerase's requirement for cellular PARylation, using the optimized viral polymerase minigenome reporter assay. For these experiments, 293T cells were used because they exhibit high transfectability for viral cDNA and minigenome plasmids [24]. Interestingly, although PARP1 itself is required for viral polymerase function, subtoxic treatment of cells with the PARylation inhibitor 4-AN (2-20uM) resulted in increased viral polymerase activity in a linear (R^2^=0.99013), dose-dependent manner (Figure 3(c)). At 20uM 4-AN treatment, polymerase activity was significantly increased (4.2±0.4 fold over DMSO vehicle,* p*<0.01, 2-tailed t test). This data suggests that cellular PARylation is directly refractive to the assembly or enzymatic activity of the influenza RdRP that synthesizes viral mRNA, reducing subsequent translation and expression of viral protein.

In human 293T cells pretreated with 4-AN (25uM) and infected with low-path, H5N1 HALo virus (MOI = 1), authentic viral polymerase activity was increased 1.8±0.4-fold over DMSO vehicle (*p*=0.06, 2-tailed t-test), as measured by a firefly luciferase reporter of influenza RdRP activity [24, 44] that is active in infected cells (Figure 3(d)). In addition, a corresponding increase in single-step growth titer of this virus in A549 cells pretreated with 4-AN (10uM) was moderate but significant (2.3±0.03 fold,* p*=0.04), analyzed by limiting dilution assay (Figure 3(e)). However, treatment with 25uM 4-AN in the single-step growth assay led to a mild but not significant increase in virus titer (1.7±0.2-fold,* p*=0.3). Taken together, the results from the minigenome assay, infected-cell polymerase reporter, and virus growth measurement indicate that pharmacological inhibition of cellular PARylation with 4-AN licensed a significant increase in influenza virus polymerase activity, leading to enhanced growth of influenza A virus in human cells.

### 3.4. Role of PARG

**Figure 4:**
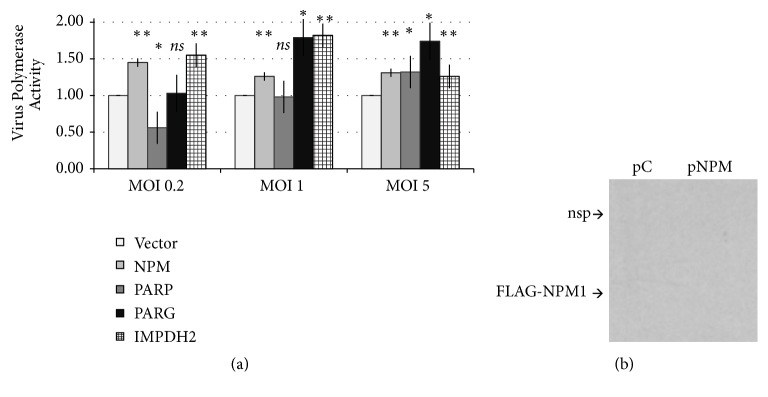
Expression of PARG, the endogenous enzyme counteracting PARP1/2 proteins, increases RdRP activity in influenza-infected cells. (a) HEK 293T cells were transfected with an RdRP reporter plasmid, and empty vector or cDNA expressing NPM, PARP1, PARG, or IMPDH2. After 24h, cells were infected with low-path, H5N1 HALo virus at MOI indicated, and viral polymerase activity was analyzed 20h.p.i. by luciferase reporter assay. All experiments were performed in independent biological duplicates with two readings per well (a total of four readings per condition). (b) Western blot with expression of FLAG-NPM1 plasmid; vector,* pC*; for PARP1 plasmid; see Figure S5B.

As a mechanism of cellular homeostasis and transcriptional control [30], the endogenous enzyme PARG removes poly-ADP ribosyl moieties from cellular macromolecules. To examine the role of PARylation in influenza virus polymerase function, we transfected 293T cells with cDNA to overexpress host factors, along with the firefly luciferase reporter to measure authentic influenza RdRP activity, and infected cells with low-path, H5N1 HALo virus (Figure 4(a)). Overexpression of NPM (Figure 4(b)), a known positive regulator of influenza polymerase led to a mild but significant (1.4-fold,* p*<0.05) increase in activity of the viral polymerase activity during infection (Figure 4(a)). A similar increase in polymerase activity for PARG overexpression was observed at a high multiplicity of infection (MOI = 1 and MOI = 5), but this was not significant (*p*<0.1), likely reflecting the incomplete efficiency of cDNA transfection. However, IMPDH2 that interacts with and supplies PARP1 and PARP2 with NAD+ substrates for enzymatic reactions [49] significantly enhances polymerase activity during infection (Figure 4(a)). These results illustrate the complexity of the IAV interaction with cellular PARylation pathways on the infected cell.

### 3.5. Localization of PARP1 and NP in the Nucleus

**Figure 5:**
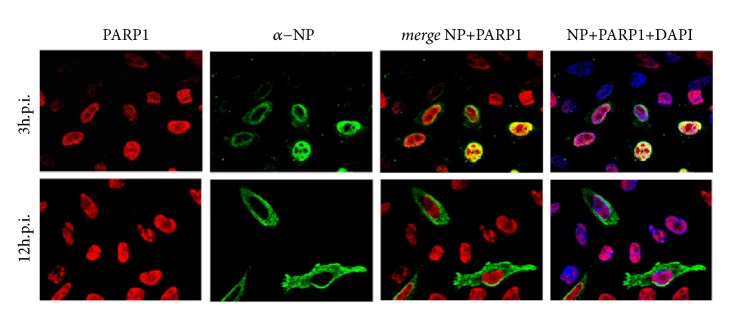
PARP1 remains in the nucleus in cells infected with influenza A virus. Human HeLa cells were infected with low-path, avian-derived influenza virus strain A/Viet Nam/1203/04 (H5N1) HALo (MOI = 0.5). Infected cell cultures were fixed for immunofluorescence 3h.p.i. and 12h.p.i. with anti-PARP (*red*) and anti-NP (*green*), and DAPI chromatin counterstaining indicating cell nuclei (*blue*).

The influenza polymerase complex and NP localize to the cell nucleus where it synthesizes viral RNA species. To better understand the interaction of PARP1 protein with the influenza polymerase and NP, we analyzed the subcellular localization of PARP1 in influenza A virus-infected cells. In resting A549 cells, PARP1 is a nuclear protein as it contains a N-terminal NLS [50]. Nuclear localization of PARP1 is maintained in cells infected with IAV even by 12h.p.i., when viral NP has translocated from nucleus to cytoplasm (Figure 5), in contrast to other host factors such as DDX17 [24] and NF90 (data not shown).

However, although PARP1 localized to the nucleus throughout infection, it only colocalized with the viral nucleoprotein early in infection in cells expressing abundant NP distributed throughout the nucleoplasm. This may correspond to an earlier phase of virus life cycle, where NP is involved primarily in viral mRNA synthesis. NP that has bound viral RNA has been reported to localize to the nuclear periphery prior export to the cytoplasm [51]. The NP accumulated at the nuclear periphery by 3h.pi. did not significantly overlap with PARP1 (Figure 5), suggesting that the phenotypes observed in knockdown of PARP1 and inhibition of PARylation may result from transient interactions between PARP1 and viral proteins and indirect effects on RdRP functions.

## 4. Discussion

### 4.1. PARP1 Is a Facilitator of IAV Infection

Poly-ADP ribosylation (PARylation) is central to cellular viability, gene expression, and metabolism and mediated by the (PARP) proteins. Small-molecule inhibition of PARP leads to disruption of DNA-damage repair (DDR) pathways and has been explored as potential anticancer chemotherapy [33, 52]. Because of its role in DDR, and potent ability to target chromatin modifiers, PARP1 was explored in the regulation of viruses [32] with a DNA genome stage in their life cycle. PARP1 functions as an antiviral protein in a preintegration step of avian retrovirus infection [53], hepatitis B virus transcription [42], and lytic replication of tumor-associated gammaherpesviruses EBV [54] and KSHV [31]. Unlike PARP1's antiviral activity observed in DNA viruses, however, we showed that PARP1 is a cofactor in the activity of the influenza A virus polymerase. In RdRP replicon (minigenome) assays and in authentic infection, PARP1 was required for optimal influenza polymerase activity, which canonically includes viral mRNA and vRNA and protein syntheses. These results are consistent with previous studies showing that PARP1 knockdown limits viral polymerase activity in a minigenome reporter assay [24] and is required to complete the virus life cycle [20, 24].

However, we found that PARP1's role in influenza infection appears to be more complex: inhibition of the PARylation enzymatic activity of PARP1 and PARP2 leads to increased influenza polymerase activity and greater replication of virus in human cells. We also found that the endogenous protein PARG, which counteracts cellular PARylation, had a similar effect to enhance influenza polymerase activity. The mechanisms by which PARP1 and PARylation function in an antiviral fashion against retroviruses, HBV, EBV, and KSHV are virus-specific; in general, PARP1 appears to act as a transcriptional repressor of DNA virus gene expression. While IAV has no DNA stage in its replication, its RdRP is highly dependent on cellular RNA polymerase II and cofactors [17–19]. Thus, it is possible that PARP1 is required for function of host cell factors that in turn normally facilitate IAV RdRP functions. Meanwhile, PARylation of PB2 and PA inhibit assembly of RdRP [37], and also PARP protein can activate antiviral pathways [32, 33, 36]. The influenza PB1 protein antagonizes one of these PARP proteins, PARP13/ZAP [37]. However, PARP1 may be cleaved and deactivated during apoptosis in IAV-infected cells [55], although this was not obvious in the experiments in this study (Figures S2A and S5). Thus, PARP1's multitude of cellular functions could both facilitate, and simultaneously inhibit, the influenza A virus polymerase and the virus life cycle through both direct and indirect mechanisms.

### 4.2. PARP1 Is Not a Strain-Specific Host Factor of IAV

The influenza polymerase minigenome reporter assay suggested that PARP1 is required for optimal function of avian, swine, and human RdRP (Figure 1(a)). The polymerase's dependence on PARP1 during authentic influenza A virus infection for synthesis of NP (Figure 2(a)) was consistent with the minigenome experiments and reported 53% reduction in viral titer in infected cells [24]. This analysis suggests that measuring polymerase activity is a useful, biosafe proxy assessment for replicative capacity and host factor dependence of highly pathogenic avian influenza virus (HPAIV) strains. Moreover, in previous studies, we found that PARP1 was also required for polymerase function for avianized revertant PB2|K627E, suggesting that the mammalian adaptive mutations of the H5N1 HPAIV polymerase are not PARP1-dependent [24]; rather, RNA binding and signaling factors such as DDX17 [24], DDX19, protein kinase C, and ANP32A/pp32 [24–27] mediate this virus-host relationship. However, the precise mechanisms of PARP1's interaction with IAV* in vivo *may vary with the many hosts and virus strains extant in nature.

### 4.3. Model of PARP1 Role in Influenza Infection

Given the interaction of PARP1 with the viral RNP [20], and evidence that PARP1 was required for RdRP function (Figure 1(a)) and weakly for viral RNA synthesis (Figure S1), a transient intranuclear interaction of PARP1 with viral polymerase and/or NP can be proposed as a mechanism. We observed that PARP1 remains in the nucleus during IAV infection (Figure 5(a)). Preliminary experiments suggest that PARP1 was pulled down by NP in the presence of viral RNA, but associated most strongly with RdRP complexes containing NP but treated with RNase to remove viral RNA (Figures S3). We observed that NPM, a binding partner of another polymerase interactor NCL [56], could also effectively displace PARP1 from interaction with NP. It could be that RNA organizes the NP and polymerase proteins in a manner that is refractory to interaction with PARP1 (Figures S3A and S3C). Consequently, our data suggests a model in which PARP1 transiently interacts with NP to facilitate polymerase function but can be displaced by other host factors as viral RNA syntheses progress in the infected cell.

In contrast, PARylation itself has an inhibitory effect on viral polymerase function (Figure 3), like with DNA viruses EBV [54] and KSHV [31]. Consistent with this model, overexpression of PARP1 cDNA weakly inhibited influenza polymerase activity (~45%) at a low MOI infection (MOI = 0.2,* p*<0.1) (Figure 4(a)). PARP1 overexpression also appeared to reduce polymerase activity in the minigenome assay, an effect that could be partly titrated away by increasing NP (Figures S4); however, results were not statistically significant (*p*<0.1). This data suggests that cellular PARylation is directly refractive to the assembly or enzymatic activity of the influenza RdRP. A study of interaction of PARP1 with influenza proteins using bimolecular fluorescent complementation [57] may shed additional light on the molecular mechanisms of this dynamic virus-host interaction.

## 5. Summary

The cellular enzyme PARP1 is a new target in for understanding diverse virus-host interactions [58]. In this study, our results have demonstrated the importance of PARP1 in influenza A virus RNA-dependent RNA polymerase function and replication. Thus, PARP1 and other proteins in the virus-host interaction network are attractive targets for deeper study of host factors that regulate influenza virus infection and pathogenesis and development of new virus-host targeted molecules as antiviral therapy against severe influenza infection.

## Data Availability

The data used to support the findings of this study are included within the supplementary information file.

## Supplementary Materials

Supplementary Materials Figure S1. Knockdown of PARP1 weakly decreases viral mRNA and vRNA syntheses. (a) Human 293T cells were treated with nontarget siRNA (lanes labelled* N*) or siRNA pool targeting PARP1 (lanes labelled* P*). Total RNA was harvested for primer extension analysis with specific primers for HA or NP viral mRNA and vRNA synthesis, as indicated, for mock-infection or 20h.p.i. after infection with low-path, avian-derived influenza virus strain A/Viet Nam/1203/04 (H5N1) HALo (MOI = 1, ref. mBio2011).* 5S*, rRNA loading control;* nsp*, nonspecific background band. (b) Viral RNA quantification by densitometry, normalized to 5S ribosomal RNA and background band. Significance of differences was estimated by unpaired, 2-tailed t-test from density histograms (*p*<0.09 for PARP1 siRNA conditions for all viral RNA species) and one-way ANOVA (*p*=0.002 for NP and HA mRNA species, and* p*=0.008 for NP and HA vRNA species). (c) Immunoblot showing PARP1 protein depletion with actin protein as internal reference. Figure S2. Expression of PARP1 protein in IAV H1N1-infected cells. (a) Human 293T cells, and cells infected with IAV (WSN, MOI = 2). (b) Human lung A549 cells infected with IAV (PR8, MOI = 0.5). In both, equivalent protein lysates were analyzed by Western blot for PARP1, viral NP, and, where shown, GAPDH control, at indicated timepoints. Figure S3. PARP1 associates with influenza A virus NP in an RNA-independent manner. (a) and (b) FLAG-tagged NP, vRNA, and polymerase constructs (vPOL: PB1, PB2, PA) were transfected into 293T cells and immunoprecipitated with anti-FLAG monoclonal (M2) antibody agarose beads (Sigma-Aldrich). Lysates were treated with RNase as indicated and Western blots probed for PARP1 with full and cleaved bands visible in 293T cells in (*b*) and NP with anti-NP antibody. (c) HA-tagged NP, vRNA, and FLAG-tagged NPM were transfected into 293T cells and lysates harvested for immunoprecipitation with anti-HA antibody agarose beads (Sigma-Aldrich) and probing for PARP1 and NP. Figure S4. Exogenous PARP1 expression inhibits influenza polymerase activity. PARP1, PARG, or vector (pC) cDNA were cotransfected with H5N1 influenza polymerase plasmids, NP, and minigenome reporter in 293T cells. IAV RdRP plasmid ratios were NP:PB1:PB2:PA of 2:2:1:2 (low NP) or 5:2:1:2 (high NP), and PARP and PARG were expressed as 100ng or 200ng of plasmid (concentration triangles). Statistical t-test analyses indicated weak (*p*<0.1) inhibition of polymerase by 100ng or 200ng PARP1 plasmid only in the low NP condition; all other pairwise* p*>0.1. All experiments were performed in biological duplicates with two readings per well. Figure S5. Exogenous PARP1 expression protects residual influenza polymerase activity from staurosporine. 200ng plasmid encoding vector (pC), or PARP1 or PARG cDNA, was cotransfected with H5N1 influenza polymerase plasmids, NP, and minigenome reporter and* Renilla* control into 293T cells and cotreated with DMSO vehicle or staurosporine (1uM). (a) Influenza polymerase minigenome activity was measured after 20 hours; or (b) wells were lysed and PARP1 and GAPDH analyzed by Western blot.
