# Supplementary figures and images for: Poly-ADP Ribosyl Polymerase 1 (PARP1) Regulates Influenza A Virus Polymerase
Source: Adv Virol. 2019 Mar 19;2019:8512363. doi: 10.1155/2019/8512363 (PMC6444269; doi:10.1155/2019/8512363)

Fig. S1

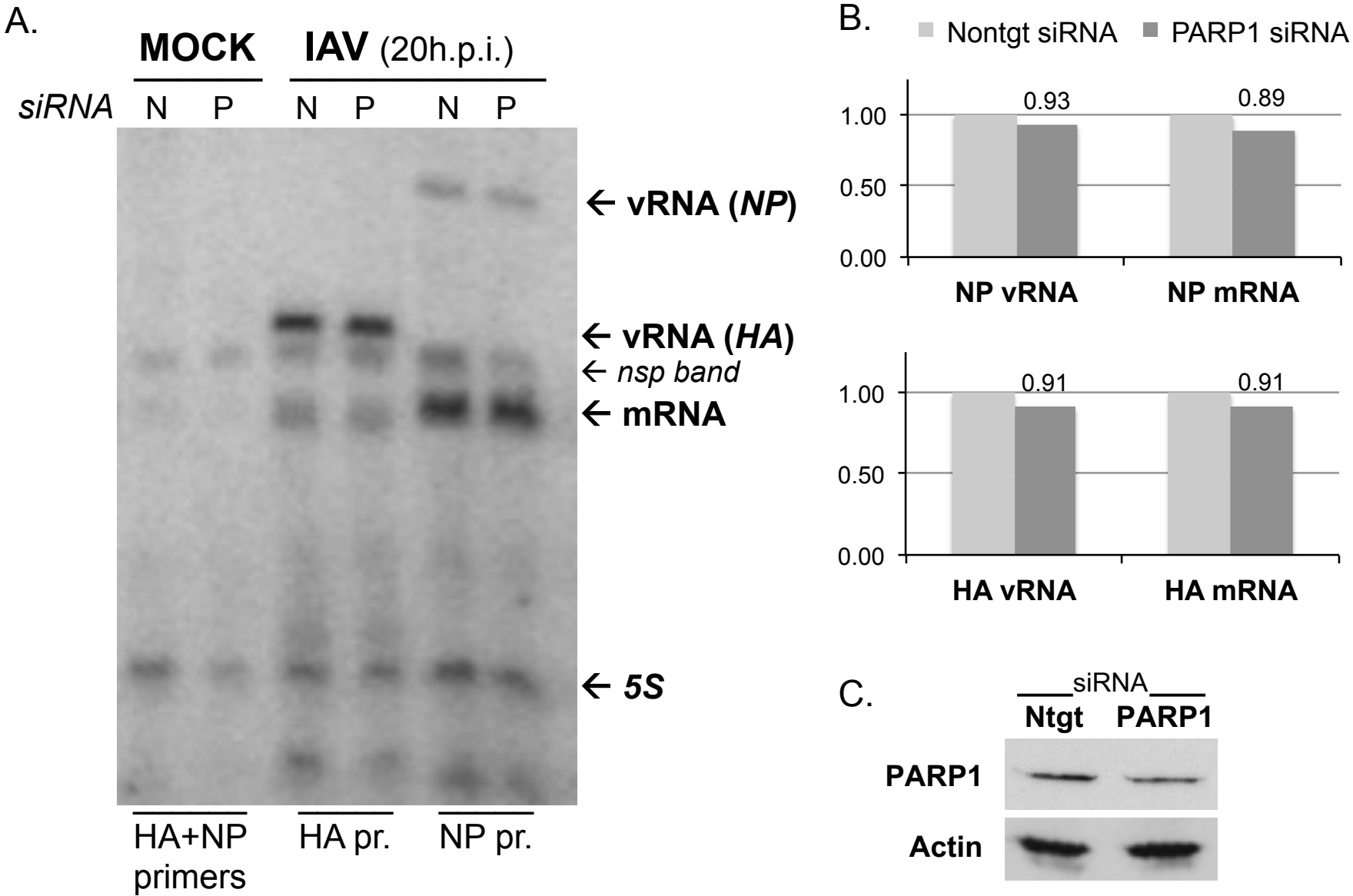

Fig. S2

A.

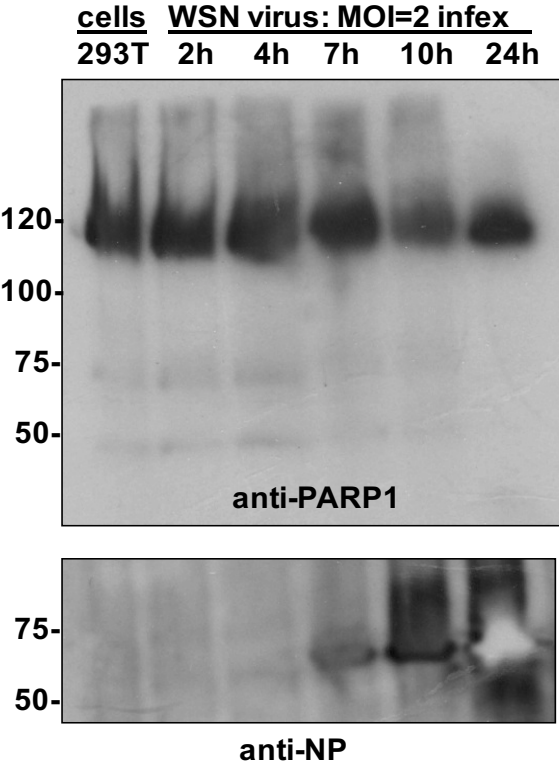

B.

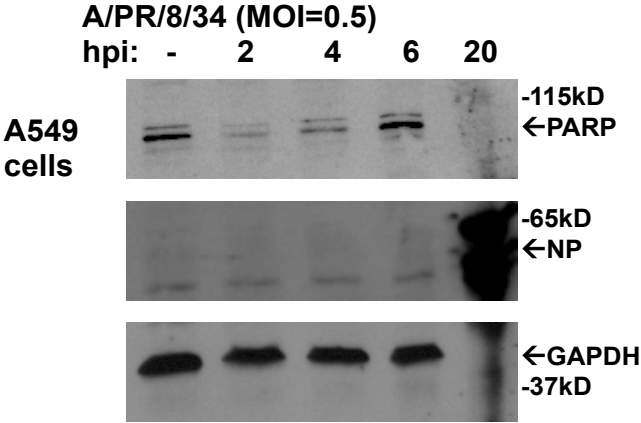

Fig. S3

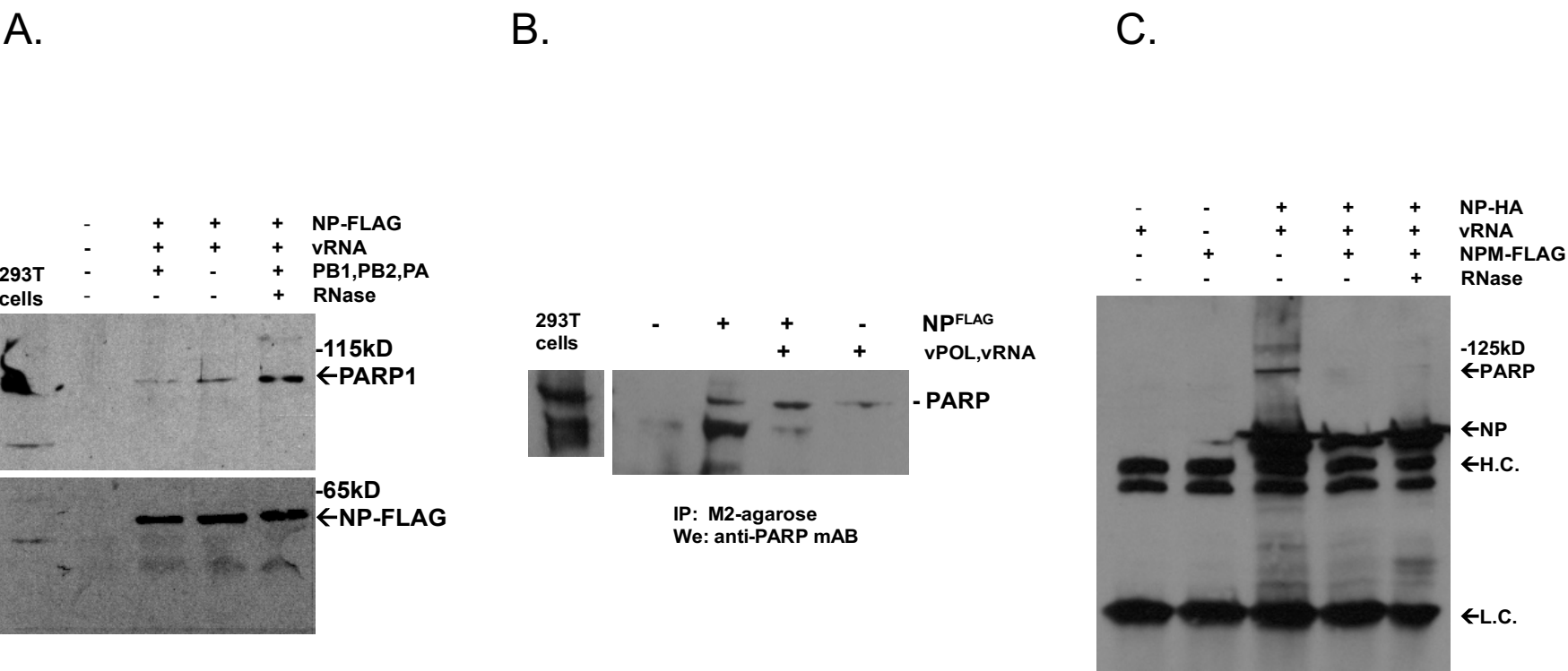

Fig. S4

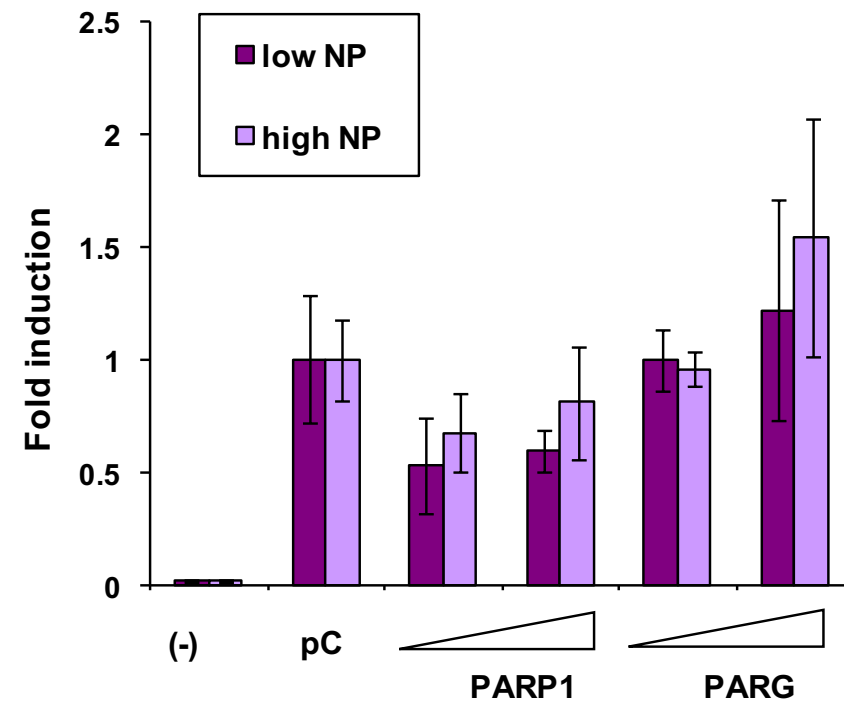

Fig. S5

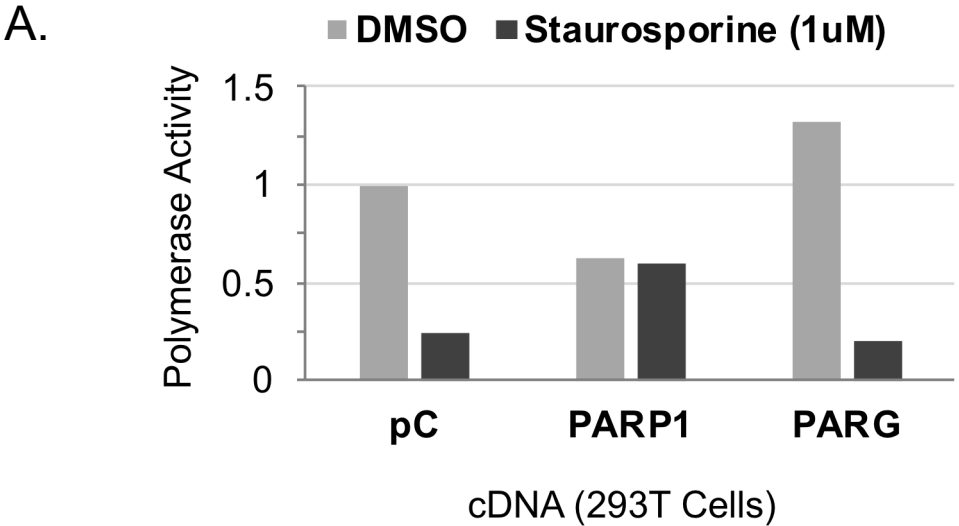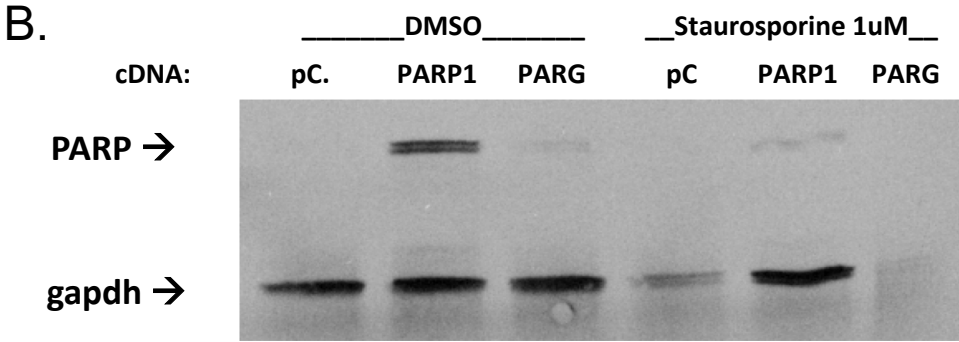

Supplement: Supplementary Materials — Figure S1. Knockdown of PARP1 weakly decreases viral mRNA and vRNA syntheses. (a) Human 293T cells were treated with nontarget siRNA (lanes labelled N) or siRNA pool targeting PARP1 (lanes labelled P). Total RNA was harvested for primer extension analysis with specific primers for HA or NP viral mRNA and vRNA synthesis, as indicated, for mock-infection or 20h.p.i. after infection with low-path, avian-derived influenza virus strain A/Viet Nam/1203/04 (H5N1) HALo (MOI = 1, ref. mBio2011). 5S, rRNA loading control; nsp, nonspecific background band. (b) Viral RNA quantification by densitometry, normalized to 5S ribosomal RNA and background band. Significance of differences was estimated by unpaired, 2-tailed t-test from density histograms (p<0.09 for PARP1 siRNA conditions for all viral RNA species) and one-way ANOVA (p=0.002 for NP and HA mRNA species, and p=0.008 for NP and HA vRNA species). (c) Immunoblot showing PARP1 protein depletion with actin protein as internal reference. Figure S2. Expression of PARP1 protein in IAV H1N1-infected cells. (a) Human 293T cells, and cells infected with IAV (WSN, MOI = 2). (b) Human lung A549 cells infected with IAV (PR8, MOI = 0.5). In both, equivalent protein lysates were analyzed by Western blot for PARP1, viral NP, and, where shown, GAPDH control, at indicated timepoints. Figure S3. PARP1 associates with influenza A virus NP in an RNA-independent manner. (a) and (b) FLAG-tagged NP, vRNA, and polymerase constructs (vPOL: PB1, PB2, PA) were transfected into 293T cells and immunoprecipitated with anti-FLAG monoclonal (M2) antibody agarose beads (Sigma-Aldrich). Lysates were treated with RNase as indicated and Western blots probed for PARP1 with full and cleaved bands visible in 293T cells in (b) and NP with anti-NP antibody. (c) HA-tagged NP, vRNA, and FLAG-tagged NPM were transfected into 293T cells and lysates harvested for immunoprecipitation with anti-HA antibody agarose beads (Sigma-Aldrich) and probing for PARP1 and NP. Figu [file 8512363.f1.pdf]
